# The Effect of the Back Surface Field on the Performance of Cu_3_SnS_4_ Thin Film Solar Cell Modeled Using SCAPS-1D Software

**DOI:** 10.3390/nano16100597

**Published:** 2026-05-13

**Authors:** Serap Yiğit Gezgin, Şilan Baturay, Shrouk E. Zaki, Hamdi Şükür Kiliç

**Affiliations:** 1 Department of Physics, Faculty of Science, Selcuk University, 42030 Selcuk, Konya, Turkey; serap.gezgin@selcuk.edu.tr; 2 Department of Physics, Faculty of Science, Dicle University, 21280 Sur, Diyarbakir, Turkey; silan@dicle.edu.tr; 3 Optics Research Group, Department of Imaging Physics, Faculty of Applied Sciences, Delft University of Technology, Mekelweg 2, 2628 CD Delft, The Netherlands; 4 Department of Nanotechnology and Advanced Materials, Graduate School of Applied and Natural Science, Selcuk University, 42030 Selcuk, Konya, Turkey; 5 Department of Metallurgy and Materials Engineering, Faculty of Engineering, University of Dokuz Eylul, 35220 Konak, Izmir, Turkey; hamdisukur.kilic@deu.edu.tr

**Keywords:** solar cell, CTS, thin film, SCASP-1D, BSF

## Abstract

In this study, the PV performance of Au/BSF/CTS/CdS/i-ZnO/ITO thin-film solar cell (TFC) structure was systematically investigated using SCAPS-1D software. The effects of several critical parameters, including interface defect density, recombination mechanisms, absorber defect density, operating temperature, parasitic resistances, and different back surface field (BSF) layers, were comprehensively analyzed. The SCAPS-1D software results reveal that the photovoltaic performance is highly sensitive to the defect density at the absorber layer interface. When the interface defect density increased from 10^12^ cm^−3^ to 10^16^ cm^−3^, the open-circuit voltage ($V_{OC}$) decreased from approximately 0.68 V to 0.45 V, while the power conversion efficiency (PCE) declined from nearly 19% to about 7%. Similarly, an increase in absorber defect density enhanced the Shockley–Read–Hall recombination rate, thereby reducing carrier lifetime and significantly deteriorating PV parameters. The influence of radiative and Auger recombination ($B_{Auger}$) processes was also examined, revealing that higher recombination coefficients lead to substantial reductions in current density and efficiency due to increased carrier losses. Furthermore, the impact of parasitic resistances was evaluated, demonstrating that decrease the series resistance from 9.5 Ω·cm^2^ to 0.5 Ω·cm^2^ increased the fill factor (FF) from about 48% to nearly 78%, while the device efficiency improved to approximately 32%. In addition to these parameters, particular emphasis was placed on the investigation of different BSF materials to enhance back contact performance. Various BSF layers, including SnS, PbS, V_2_O_5_, and Sb_2_S_3_, were examined to improve band alignment and suppress minority carrier recombination at the rear interface. Among these materials, the SnS BSF layer provided the most favorable band alignment with the CTS absorber, leading to a notable improvement in PV parameters and increasing the efficiency to approximately 25%. Overall, the results demonstrate that optimizing defect densities, recombination mechanisms, parasitic resistances, and especially the selection of appropriate BSF materials plays a crucial role in improving the performance of CTS-based TFCs.

## 1. Introduction

In recent years, the rapid increase in global energy demand, along with the limited availability of conventional energy resources, has intensified exceptional interest in renewable energy solutions, particularly focusing on the design and fabrication of PV cells that can exchange solar energy into electrical power. In order to accelerate the advancement of PV technologies, minimizing production costs and material consumption is crucial. In this regard, among the most widely studied polycrystalline TFCs, CdTe and Cu(In,Ga)Se_2_ have demonstrated high power conversion effectiveness of approximately 23–25% and 27%, respectively [1,2,3]. Nevertheless, the absorber layers employed in these structures incorporate elements such as tellurium (Te), indium (In), gallium (Ga), and selenium (Se), which are not only scarce and limited in availability but also pose significant toxicity concerns (e.g., Cd, Se, and Te). This challenge has prompted researchers to investigate alternative materials. In this context, substituting environmentally friendly elements, such as In, Se, and Ga, as well as cost-effective and earth-abundant elements like copper (Cu), tin (Sn), zinc (Zn), and sulfur (S), has enabled the development of Cu_2_ZnSnS_4_ (CZTS) TFCs, which is a novel and highly promising absorber layer. Even though CZTS thin-film solar cells have achieved a maximum PCE of around 15% [4], synthesizing phase-pure CZTS without the formation of secondary phases such as Cu–Sn-S/Cu-S, Sn–S or Zn–S remains an important challenge because of the complex nature of its phase diagram and the difficulty in obtaining a uniform single-phase material. In addition, the suppression and identification of secondary phases pose significant difficulties. While various chemical-etching techniques have been developed to eliminate these phases, they are generally effective only at the surface, leaving secondary phases embedded deeper within the material largely unaffected [5,6]. On the other hand, controlling the zinc content during the deposition of CZTS samples is mainly challenging because of the relatively high vapor pressure of Zn related to other constituent elements. Furthermore, Zn-related defects are generally deep-level and are considered among the main factors limiting the performance of CZTS thin-film solar cells [7,8]. Additionally, ZnS, an *n*-type semiconductor, may form at various locations within CZTS samples, thereby reducing the carrier concentration. To mitigate the challenges described above, it is more practical to fabricate and investigate Cu–Sn–S samples with a ternary structure that excludes Zn. Compared to CZTS, Cu–Sn–S samples are easier to control in terms of both elemental composition and defect formation, owing to the fact that they are composed of only three environmentally benign elements. The development and fabrication of Cu–Sn–S ternary compounds have garnered significant interest among researchers, not only because of their environmental friendliness and cost-effectiveness but also due to their natural abundance and potential for stable optoelectronic properties. This ternary system is composed of distinct phases: Cu_2_SnS_3_, Cu_3_SnS_4_, and Cu_4_SnS_4_. Previous studies have demonstrated that each of these phases possesses favorable optoelectronic properties, making them suitable candidates for use as absorber layers in PV applications [9,10]. Moreover, their high absorption coefficients and tunable energy gaps further enhance their potential for efficient conversion of solar energy to electric power. Currently, Cu_3_SnS_4_ (CTS) semiconductors stimulate some increase in attention as important members of I–IV–VI groups with relatively narrow energy band gaps. The environmentally friendly and low-cost nature of CTS has made it an appealing material for a wide range of PV applications. Depending on the crystal structure, CTS exhibits different optoelectronic properties: samples with cubic and monoclinic structures have band gaps in the range of 0.9–1.0 eV, whereas tetragonal and orthorhombic phases display larger band gaps between 1.3 and 1.6 eV [11,12]. These variations in crystal structure and corresponding band gaps highlight the tunability of CTS for specific solar cell applications. The primary application of CTS is in solar cells; however, CTS nanoparticles (NPs) obtained via chemical techniques have also been explored for photocatalytic applications, Na-ion batteries, gas sensing, photocatalytic hydrogen (H) production, and photonic devices, owing to their porous microstructure and high surface area [12,13,14,15,16].

In light of the properties discussed above, CTS stands out as a highly promising absorber material for the next generation of TFCs. A major focus in current PV research and development is to create solar cells that are commercially viable by satisfying three essential requirements: high energy conversion efficiency, cost-effectiveness, and reliable operational stability. Decreasing the thickness of CTS absorber layer can substantially reduce the quantity of material needed, leading to lower manufacturing costs [12,17]. Additionally, optimizing the thickness of different layers throughout the solar cell structure not only cuts down on production expenses, energy consumption, and fabrication time but also ensures more efficient use of materials overall.

In 2025, Manimozhi et al. [18] reported that a DSSC employing a CTS/graphene (50 mg) nanocomposite as the counter electrode achieved a PCE of 10.21% through a simple one-pot solvothermal synthesis. Liu et al. [19] demonstrated that a 40 nm CTS counter electrode deposited on FTO by DC magnetron sputtering technique with a ceramic target reached a maximum PCE of 7.75%, comparable to commercial platinum electrodes. In 2017, Chen et al. [20] synthesized a tetragonal CTS sample on FTO substrate via solvothermal sulfurization of a Cu–Sn precursor, and the resulting CTS/FTO counter electrode exhibited a PCE of 7.80%, surpassing that of a Pt electrode (6.52%). However, the solvothermal technique requires a long reaction time (~20 h), which limits its cost-effectiveness for DSSC fabrication. Despite these advances, the maximum PCE of CTS-based TFCs remains approximately three times lower than that of other second-generation thin-film technologies, indicating the need for further research to enhance their efficiency.

With the goal of advancing CTS-based solar cells toward commercial viability, researchers have focused on developing an improved device architecture consisting of ITO/ZnO/CdS/CTS/BSF/Au metal contact, in which ITO functions as a transparent conductive layer, ZnO acts as an electron transport layer, CdS serves as an effective window layer that facilitates light absorption while minimizing recombination losses, CTS absorber provides a low-cost, environmentally friendly, and earth-abundant alternative to traditional materials with tunable band gaps suitable for efficient PV conversion, and the BSF and Au contact are designed to enhance carrier collection and reduce energy loss, with considerable effort devoted to optimizing the thickness, composition, and interface properties of each layer to maximize overall PCE [21,22,23]. Accordingly, the primary objective of this study is to explore a novel CdS/CTS thin-film solar cell configuration aimed at enhancing PCE while reducing production costs through minimizing material layer thicknesses, and a new device architecture comprising Au/BSF/CTS/*n*-CdS/i-ZnO/ITO has been proposed, with multiple strategies being investigated to optimize performance through advanced cell design. Numerical simulation provides a powerful approach for investigating how changes in material properties affect the performance of solar cells, allowing researchers to evaluate design feasibility, optimize structural parameters, and predict device behavior prior to experimental fabrication, and in this study, simulations were performed using SCAPS-1D platform to examine the influence of the thickness of monoclinic CTS absorber layer and charge carrier concentration on key metrics, with the impact of varying the buffer layer thickness analyzed to determine the optimal configuration for establishing an efficient interface with the CTS absorber and to evaluate its contribution to overall cell efficiency, while essential output parameters, including $V_{OC}$, short-circuit current density ($J_{SC}$), FF, and PCE, were systematically recorded and analyzed to characterize the proposed device structures’ performance. Moreover, in this study, SCAPS-1D simulations also considered critical parameters such as interface defect density, radiative and $B_{Auger}$ coefficients, and the characteristics of various BSF layers to evaluate their influence on the PV performance of the CTS-based solar cell. This comprehensive approach ensures that the interplay between material properties, layer thicknesses, and interface engineering is fully captured, providing deeper insight into the design of high-efficiency CTS TFCs.

In addition to the material-level advantages discussed above, CTS-based solar cells are also highly compatible with sustainable energy systems. Owing to their composition of earth-abundant, low-toxicity elements and their compatibility with scalable thin-film fabrication techniques, as well as CTS absorbers, provide a promising route toward cost-effective large-scale photovoltaic deployment. However, it is important to note that many existing studies, including device-oriented investigations, predominantly focus on material and device optimization, often lacking sufficient connection to broader energy system considerations such as scalability, system integration, and long-term sustainability. Addressing this gap is essential for evaluating the real-world applicability of CTS-based technologies. In this context, recent studies emphasize the integration of PV technologies into multi-energy systems, where electricity generation is synergistically combined with thermal energy management and hydrogen production/storage to improve overall system efficiency and flexibility. In particular, risk-aware optimization frameworks for hydrogen-oriented energy systems highlight the critical role of PV technologies in enabling low-carbon and resilient energy infrastructures [24]. Furthermore, CTS materials exhibit potential for photoelectrochemical hydrogen generation, extending their functionality beyond conventional PV applications. Recent advances in functional materials also provide new opportunities to enhance CTS device performance. For instance, bioinspired photothermal superhydrophobic metamaterials have demonstrated improved photo/electro–thermal conversion and anti-/de-icing capabilities [25,26], while plasmonic metamaterials with optical valve characteristics enable selective spectral absorption for advanced light management [27]. Additionally, the development of highly conductive PEDOT:PSS hole transport layers has significantly improved interfacial charge transport in emerging PV systems [28]. These interdisciplinary developments suggest that CTS-based solar cells can be further optimized in terms of light harvesting, carrier transport, and environmental stability, reinforcing their potential role not only as efficient photovoltaic absorbers but also as key components in hybrid, hydrogen-integrated, and low-carbon multi-energy systems.

In this study, the implementation of SnS, PbS, V_2_O_5_, and Sb_2_S_3_ as BSF layers to effectively suppress the recombination of minority charge carriers at the rear contact region while enhancing carrier collection within the depletion region. This strategic band alignment and back interface engineering result in a significant improvement in the overall photovoltaic performance of the solar cell. In addition, the comprehensive optimization framework, in which critical device parameters, including interface defect density, radiative and $B_{Auger}$ within the absorber layer, bulk defect density, series and shunt resistances, and operating temperature are systematically varied. Based on this parametric analysis, the study determines the attainable lower and upper bounds of the PV parameters, thereby providing a more realistic performance window and deeper insight into the operational limits and stability of the proposed device structure.

## 2. Characterization of Cu_3_SnS_4_ Thin Film

The XRD results of the previously obtained CTS-1 for annealed at 30 sccm sulphur flux (15 min), CTS-2 for annealed at 30 sccm sulphur flux (30 min), CTS-3 for annealed at 40 sccm sulphur flux (15 min), and CTS-4 for annealed at 40 sccm sulphur flux (30 min) samples reveal that the dominant phase in all samples is orthorhombic Cu_3_SnS_4_ with a pronounced preferential orientation along the (222) plane [22].

**Figure 1:**
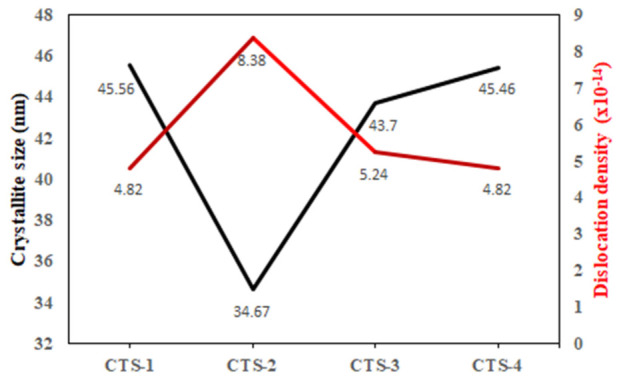
The crystalline size and dislocation density change of CTS thin films.

The crystallite sizes calculated from the XRD data for the (222) plane range from approximately 34.7 to 45.6 nm, while the dislocation density varies between 4.82 × 10^14^ and 8.32 × 10^14^ m^−2^. The decrease in crystallite size accompanied by an increase in dislocation density indicates an increase in lattice imperfections and internal strain. This behavior is associated with the higher sulfur flux and prolonged annealing time, which limit grain growth and promote defect formation within the crystal structure. The variations in crystallite size and dislocation density are presented in Figure 1.

**Figure 2:**
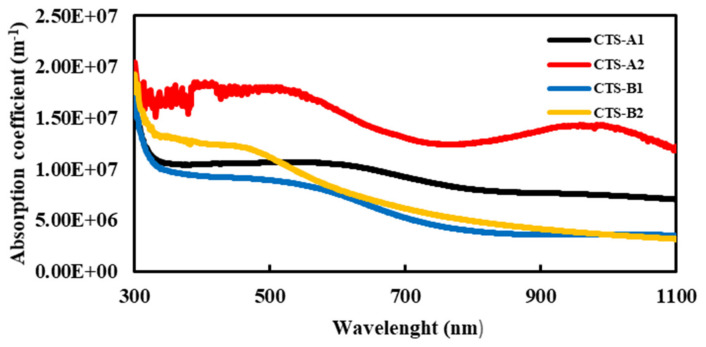
The absorption coefficient of CTS thin films.

The optical behavior of the previously reported CTS-1, CTS-2, CTS-3, and CTS-4 samples demonstrates a strong dependence on sulfur flow rate and annealing conditions (Figure 2).

In the visible region, the absorption coefficient reaches values on the order of 10^7^ m^−1^, with CTS-2 showing the most pronounced absorption, while samples prepared under higher sulfur flux exhibit relatively reduced values. This trend can be associated with sulfur-induced compositional deviations and the emergence of secondary phases (e.g., Cu_2_S, SnS_2_), which disturb the optical uniformity of the Cu_3_SnS_4_ matrix and weaken its effective light-harvesting capability. Despite this, all films maintain sufficiently high absorption coefficients (>10^6^ m^−1^), confirming their potential for optoelectronic applications.

**Figure 3:**
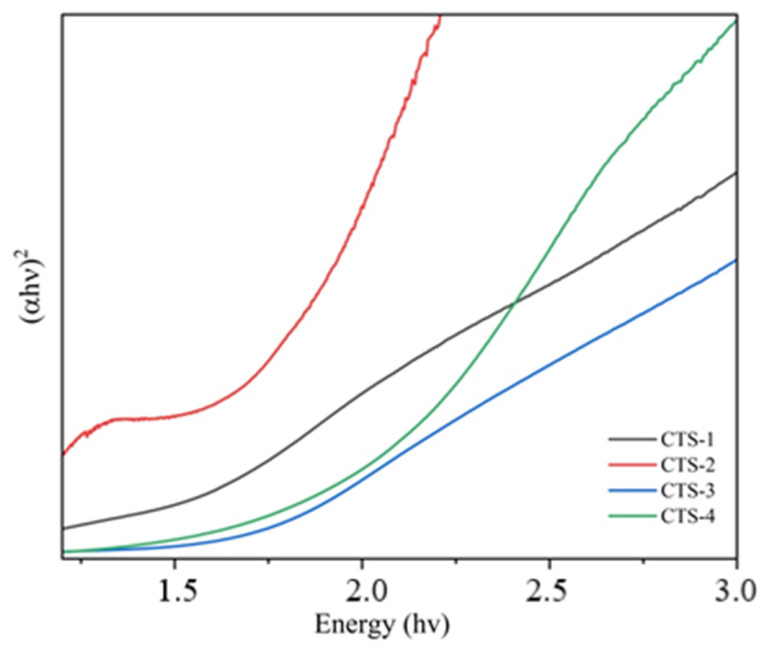
The energy band gap of CTS thin films.

The band gap energies, extracted from Tauc analysis (Figure 3), vary between 1.36 and 2.10 eV, showing a systematic increase with increasing sulfur content. This behavior is attributed to changes in the electronic structure arising from lattice imperfections, phase inhomogeneity, and reduced crystallinity, all of which influence the optical transitions in the material.

**Figure 4:**
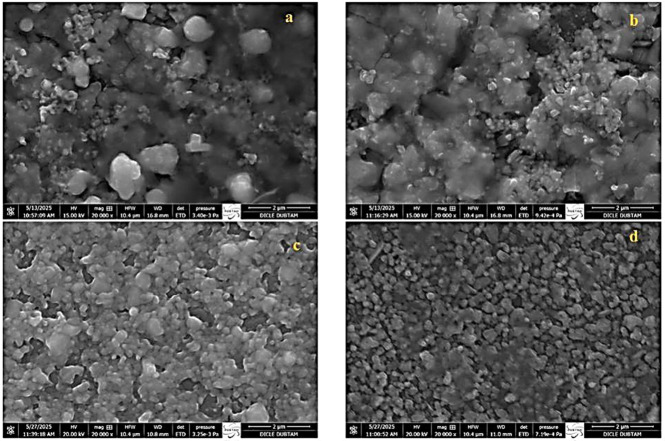
SEM images of (**a**) CTS-1, (**b**) CTS-2, (**c**) CTS-3, and (**d**) CTS-4 thin films.

The SEM image (Figure 4) shows that the CTS film annealed at 550 °C exhibits a homogeneous and well-adhered surface with nearly spherical Cu_3_SnS_4_ grains. Compared to CTS-1, prolonged annealing leads to a denser morphology composed of loosely packed nanocrystals, confirming the polycrystalline nature of the films. Changes in sulfur flux modify grain structure and surface coverage, indicating that sulfur content plays a key role in determining the film morphology and related properties [22].

**Figure 5:**
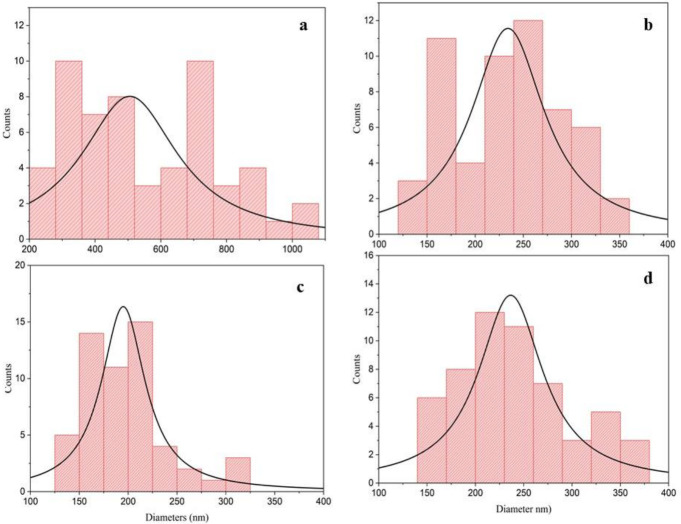
Histogram of (**a**) CTS-1, (**b**) CTS-2, (**c**) CTS-3, and (**d**) CTS-4 thin films.

The grain size distribution obtained from the histogram analysis is presented in Figure 5. The average grain sizes were determined to be approximately 480 nm for CTS-1, 240 nm for CTS-2, 190 nm for CTS-3, and 240 nm for CTS-4. These values are consistent with the SEM observations (Figure 4), where noticeable variations in grain size and surface morphology are evident among the samples. The reduction in grain size, particularly for CTS-3, can be attributed to changes in annealing conditions and sulfur content, which influence nucleation and growth mechanisms during film formation.

## 3. The Modelling of Au/BSF/Cu_3_SnS_4_/CdS/i-ZnO/ITO Thin Film Solar Cell by SCAPS-1D Software

In our earlier work, Cu_3_SnS_4_ (CTS) thin films were successfully produced by the spin coating method, and the influence of the sulphur flow rate, together with annealing time on their structural, optical, crystallographic, and electrical characteristics, was examined in detail. X-ray diffraction measurements indicated that the films predominantly crystallized in the orthorhombic CTS phase with a preferred (222) orientation, while the peak intensities were found to be strongly dependent on the annealing parameters. For samples annealed at 550 °C, the calculated crystallite sizes were within the range of approximately 34.67–45.56 nm. In addition, the dislocation density values were estimated to vary between 4.82 × 10^14^ m^−2^ and 8.32 × 10^14^ m^−2^, whereas the microstrain values were determined to be in the range of 0.32 × 10^−4^–0.42 × 10^−4^. Raman spectroscopy analysis revealed the existence of secondary phases such as SnS and Cu_2_SnS_3_, characterized by dominant vibrational modes around 228 cm^−1^. Surface investigations performed by atomic force microscopy demonstrated that the average roughness values of the samples were between 32.37 nm and 50.50 nm, respectively. Scanning electron microscopy (SEM) images further confirmed a relatively uniform surface morphology composed mainly of spherical grains. Photoluminescence spectra exhibited emission features near 1.41 eV and 1.80 eV together with broad emission bands centered at approximately 549 nm, 567 nm, 689.42 nm, and 882.6 nm. Particularly, the peaks observed at 549 nm and 567 nm in the green spectral region were attributed to intrinsic defect levels associated with Cu, Sn, or S atoms. The optical band gap values were determined to vary between 1.36 eV and 2.10 eV depending on the sample conditions, suggesting the possible contribution of secondary phases. Moreover, Hall effect measurements confirmed that CTS film exhibits *p*-type conductivity with carrier concentrations on the order of 10^17^–10^18^ cm^−3^, while carrier mobility values ranged from 54.90 to 231.18 cm^2^ V^−1^ s^−1^, according to the applied annealing conditions [22].

A simulation tool is required to assess the performance of solar cells fabricated through experimental methods to model the potential behaviour of a solar cell by integrating the produced semiconductor thin films [29]. Simulation software such as SCAPS-1D, SILVACO, AMPS-1D, and ATLAS are employed to evaluate and validate the theoretical efficiency of solar cells [30,31]. SCAPS-1D is utilized to compute the efficiency of solar cells comprising multiple layers, employing an approach that deviates from traditional methodologies.

**Figure 6:**
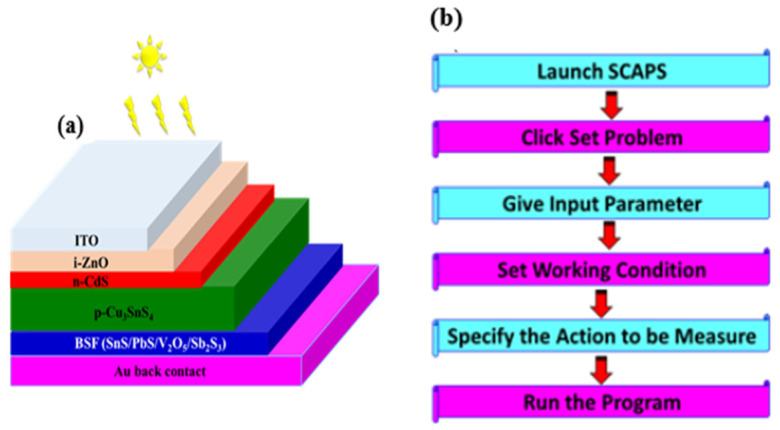
(**a**) The CTS solar cell diagram with BSF layer and (**b**) SCAPS-1D software work steps.

In this investigation, a solar cell structure consisting of Au/BSF/CTS/*n*-CdS/i-ZnO/ITO was modelled using the SCAPS-1D simulation tool, with the CTS layer serving as the absorber material. SCAPS-1D numerically resolves the fundamental semiconductor transport equations, including the coupled Poisson and carrier continuity equations, which define charge carrier dynamics within the material system. SCAPS-1D simulation platform enables systematic parameterization and grading of input variables, including recombination mechanisms, carrier polarity, bulk and interface defect states, defect energy distributions (Gaussian, discrete single-level, uniform, or hybrid configurations), and contact boundary conditions (flat-band or work-function-defined), as well as optical characteristics such as direct photo-generation processes [32]. Figure 6a,b illustrate the fundamental device configuration of the solar cell and SCAPS-1D software work steps, respectively. The absorption coefficient data file, corresponding to the CTS thin film, was incorporated into the simulation software as an input parameter. The specific physical parameters associated with the constituent layers of the CTS thin-film solar cell are summarized in Table 1. The experimentally produced CTS thin film thickness, band gap, mobility of electrons/hole charges, and density of acceptor are experimental data in Table 1. The physical parameters of the other layers are taken from the literature and referenced.

The software computes PV parameters by solving the following set of equations (Equation (1)):

(1) $$\frac{d\gamma }{dx}=-\frac{d^{2}\Psi }{dx^{2}}=\frac{q}{\varepsilon }\left[p\left(x\right)-n\left(x\right)+N_{D}^{+}\left(x\right)-N_{A}^{-}+p_{t}\left(x\right)-n_{t}\left(x\right)\right]$$

Ψ  denotes the electrostatic potential, ε represents the dielectric permittivity, q stands for the elementary charge, n and p indicate the electron and hole concentrations, respectively, $N_{A}^{-}$ corresponds to the ionized acceptor density, $N_{D}^{+}$ refers to the ionized donor density, $p_{t}$ and $n_{t}$ indicate the densities of hole and electron trap states, respectively, and x signifies the spatial coordinate [32].

Continuity equations for holes (Equation (2)) and electrons (Equation (3)) [33]:

(2) $$\frac{dp_{n}}{dt}=G_{p}-\frac{p_{n}-p_{n0}}{\tau_{p}}+p_{n}\mu_{p}\frac{dE}{dx}+\mu_{p}E\frac{dP_{n}}{dx}+D_{p}\frac{d^{2}p_{n}}{dx^{2}}$$

(3) $$\frac{dp_{p}}{dt}=G_{n}-\frac{n_{p}-n_{p0}}{\tau_{n}}+n_{p}\mu_{n}\frac{dE}{dx}+\mu_{n}E\frac{dn_{p}}{dx}+D_{n}\frac{d^{2}n_{p}}{dx^{2}}$$

$G_{n}$ and $G_{p}$ denote the rates at which electrons and holes are generated, while $n_{p}$ and $p_{n}$ represent the electron and hole concentrations within the p and n type regions, respectively. The equilibrium values of the hole concentration in the p-type region and the electron concentration in the n-type region are represented by $n_{p0}$ and $p_{n0}$, respectively. The lifetimes of electrons and holes are characterized by $\tau_{n}$ and $\tau_{p}$, while the mobilities of electrons and holes are indicated by $\mu_{n}$ and $\mu_{p}$, respectively. E denotes the electric field, while $D_{n}$ and $D_{p}$ represent the diffusion coefficients associated with electrons and holes, respectively. The transport of charge carriers due to drift and diffusion processes for electrons and holes is expressed in Equations (4) and (5):

(4) $$J_{n}\;\left(x\right)=qn\mu_{n}E+qD_{n}\frac{dn}{dx}=n\mu_{n}\frac{dE_{Fn}}{dx}$$

(5) $$J_{p}\;\left(x\right)=qp\mu_{p}E-qD_{p}\frac{dp}{dx}=n\mu_{p}\frac{dE_{Fp}}{dx}$$

$E_{Fp}$ and $E_{Fn}$ correspond to the quasi-Fermi levels for holes and electrons, respectively.

**Table 1:** Physical characteristics of the layers in the simulated Cu_3_SnS_4_ solar cell.

Layers for Solar Cells	ITO [34]	i-ZnO [18]	CdS [35]	CTS [22,36]	SnS [37]	PbS [38]	V_2_O_5_ [39]	Sb_2_S_3_ [40]
Energy Gap (eV)	3.3	3.3	2.4	1.36 (exp.)	1.3	1.3	2.2	1.62
Affinity of electron (eV)	4.6	4.6	4.2	4.7	4.2	4.2	3.4	3.7
The permittivity of dielectric	9	9	10	10	12.5	9	10	7.08
CB effective density (cm^−3^)	2.2 × 10^18^	2.2 × 10^18^	2.2 × 10^18^	2.2 × 10^18^	1.0 × 10^19^	1.0 × 10^19^	9.2 × 10^17^	2.0 × 10^19^
VB effective density (cm^−3^)	1.8 × 10^19^	1.8 × 10^19^	1.8 × 10^19^	1.8 × 10^19^	4.13 × 10^19^	4.13 × 10^19^	5.0 × 10^18^	1 × 10^19^
Thermal velocity of electron/Hole (cm/s)	1.0 × 10^7^	1.0 × 10^7^	1.0 × 10^7^	1.0 × 10^7^	1.0 × 10^7^	1.0 × 10^7^	1.0 × 10^7^	1.0 × 10^7^
Mobility of electron/Hole (cm^2^/Vs)	100/25	100/25	100/25	231.19/20 (exp.)	25/100	100/38	320/40	9.8/10
Density of shallow donor (cm^−3^)	1.0 × 10^20^	1.0 × 10^5^	1.0 × 10^18^	0	0	0	0	0
Density of shallow acceptor (cm^−3^)	0	0	0	3.13 × 10^17^ (exp.)	1.0 × 10^18^	1.0 × 10^17^	1.0 × 10^18^	1.0 × 10^15^
Thickness of film (nm)	100	100	50	857 (exp.)	Variable	Variable	Variable	Variable

### 3.1. The Influence of the Interface Defect Density

**Figure 7:**
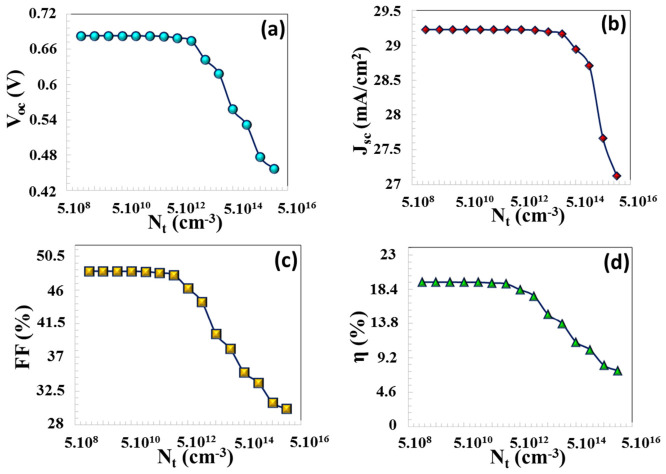
(**a**) $V_{OC}$, (**b**) $J_{SC}$, (**c**) FF, (**d**) η  PV parameters depending on the interface defect density $N_{t}$.

In solar cells, band gap mismatch, band slope mismatch, crystal defects, crystal dislocations, grain boundaries, pinholes, microscopic cracks, and stress formations due to differences in thermal expansion between two semiconductors, oxide layer formation, and sagging bond formations lead to the formation of interface defects and the recombination of photo-excited charge carriers [41]. An elevated density of interface defects leads to a reduction in the overall performance of the solar cell. The presence of interfacial defect states creates localized trapping sites that function as recombination centers [42]. An increased defect density results in a greater number of these regions, which subsequently trap more charge carriers, particularly electrons. Moreover, interfacial defects can exacerbate series resistance, leading to a significant reduction in the device’s efficiency. When the interface defect density is increased from 1 × 10^9^ cm^−3^ to 1 × 10^12^ cm^−3^, there is almost no significant change in PV parameters, which indicates that the system operates in a defect-tolerant regime in this region. This means that the trap levels at the interface are sufficiently low and carrier lifetimes are not limited by Shockley–Read–Hall (SRH) recombination. As $N_{t}\;$ increased from 1 × 10^12^ cm^−3^ to 1 × 10^16^ cm^−3^, a corresponding decline was observed in $J_{SC}$, $V_{OC}$, FF, and η, which decreased from 29.22 mA/cm^2^ to 27.12 mA/cm^2^, from 0.6820 V to 0.456 V, from 48.03% to 30.14%, and from 19.14% to 7.47%, respectively (in Figure 7). Increased interface trap density significantly increases the SRH recombination rate by creating intermediate energy levels in the semiconductor band gap, which explains the significant decrease in *V_oc_* in particular. Traps disrupt the transport path of charge carriers, reducing the diffusion length and collection probability, which leads to a decrease in *J_sc_*. Charge accumulation and trap filling at the interface disrupt the local electric field distribution, weakening band bending, which indirectly leads to a deterioration of the FF and series resistance behavior.

### 3.2. The Recombination and Generation Processes

#### 3.2.1. The Effect of the Radiative Recombination

A photon is emitted when an electron transitions from the conduction band (CB) to the valence band (VB) through a radiative recombination process. This process, where the electron and hole recombine, is referred to as radiative recombination. The $B_{r}$ is mathematically represented by Equation (6) [42]:

(6) $$B_{r}=\frac{1}{\tau_{n\;or\;p,rad}N_{A\;or\;D}}$$

**Figure 8:**
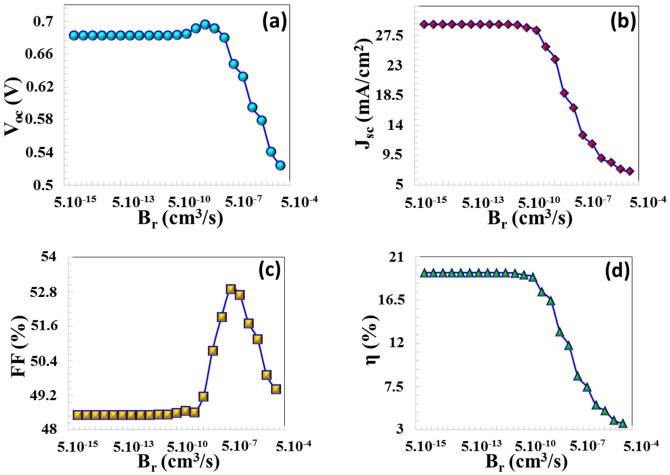
(**a**) $V_{OC}$, (**b**) $J_{SC}$, (**c**) FF, (**d**) η PV parameters as a function of the radiative recombination coefficient ($B_{r}$).

The radiative recombination coefficient, $B_{r}$ ($\frac{{cm}^{3}}{s}$), is associated with the radiative lifetimes of the electron and hole carriers, denoted as $\tau_{n,rad}\;(s)$ and $\tau_{p,rad}\;(s)$, respectively. In this work, the efficiency of CTS TFCs remained unaffected as *B_r_* varied between 1 × 10^−15^ cm^3^/s and 5 × 10^−10^ cm^3^/s. This indicates that the device is insensitive to radiative processes in terms of its recombination mechanism in this region, and performance can be largely controlled by the SRH or transport-limited region. However, when *B_r_* is arisen from 5 × 10^−10^ cm^3^/s to 1 × 10^−4^ cm^3^/s, PV parameters of the TFC, it experiences an important decrease, with $V_{OC}$ dropping from 0.684 V to 0.524 V, $J_{SC}$ decreasing from 28.75 mA/cm^2^ to 7.06 mA/cm^2^, and efficiency falling from 19.12% to 3.66%, as shown in Figure 8a–d. An enhancement in $B_{r}$ value leads to a reduction in the carrier lifetime or carrier density, which in turn results in a decline in the solar cell’s performance, as stated in Equation (6). This transition can be explained by the rapid decrease in carrier lifetime due to the *τ_rad_* ∝ 1/*B_r_* relationship, and, consequently, the weakening of the quasi-Fermi level separation, which directly leads to a decrease in *V_oc_*. At the same time, increased radiative recombination leads to a notable decrease in *J_sc_*, especially as carriers produced in the depletion region are destroyed before they can be transported within the solar cell. The deterioration in FF shows that increased recombination not only reduces the number of carriers but also weakens the internal electric field distribution and disrupts carrier transport. As a result, the device exceeds a critical threshold point in terms of production–recombination balance, and the efficiency rapidly decreases.

**Figure 9:**
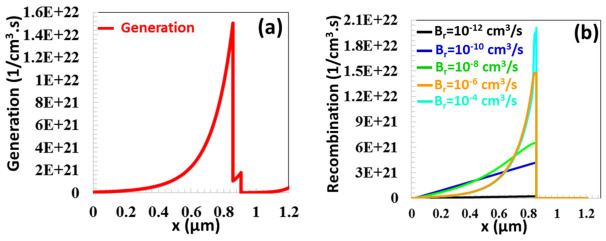
(**a**) Generation and recombination rate as a function of position (x) for the CTS TFC (**b**) at different radiative recombination coefficients ($B_{r}$) ranging from 10^−12^ cm^3^/s to 10^−4^ cm^3^/s.

In TFCs, a high concentration of photo-excited charge carriers, approximately 1.5 × 10^22^ (1/cm^3^·s), is generated in the vicinity of the depletion region of the p-type absorber layer, located at a distance of x = 0.857 μm, as demonstrated in Figure 9a. Due to the reduction in light absorption from the depletion region to the back contact, the rate of charge carrier generation declines to 8.89 × 10^19^ (1/cm^3^·s) at x=0 μm. As the radiative recombination coefficient $B_{r}$ was raised from 1 × 10^−12^ cm^3^/s to 5 × 10^−10^ cm^3^/s, the rate of radiative recombination near the depletion region increased from 2.14 × 10^20^ (1/cm^3^·s) to 4.15 × 10^21^ (1/cm^3^·s). After *B_r_* = 5 × 10^−10^ cm^3^/s (ranging from 1 × 10^−9^ cm^3^/s to 1 × 10^−4^ cm^3^/s), PV performance broke down considerably as the recombination rate surpassed the charge generation rate, as demonstrated in Figure 9b.

#### 3.2.2. The Effect of the Auger Electron Recombination

The holes and electrons recombine within the absorber layer, releasing energy during the process. This energy is subsequently transferred to the charge carriers, enabling them to transition into higher energy states without the emission of radiation. The phenomenon referred to as Auger recombination is a non-radiative process. The $B_{Auger}$ coefficient is defined by Equation (7):

(7) $$B_{Auger,\;n\;or\;p}=\frac{1}{\tau_{n\;or\;p,rad}N_{A\;or\;D}^{2}}$$

**Figure 10:**
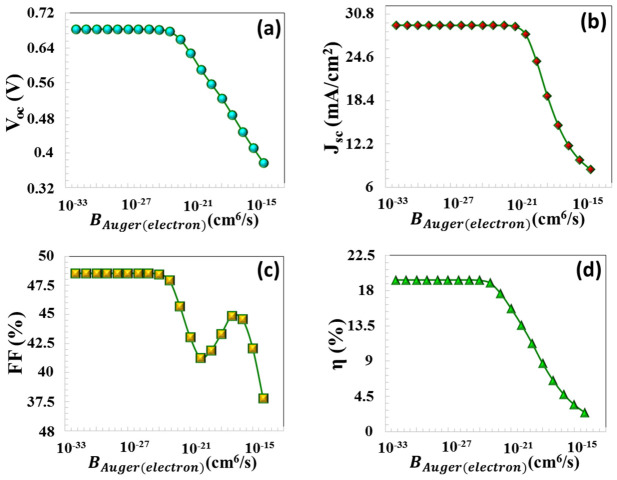
(**a**) $V_{OC}$, (**b**) $J_{SC}$, (**c**) FF, (**d**) η PV parameters as a function of Auger electron capture coefficient.

In this study, PV parameters were evaluated as a function of the $B_{Auger}\;$ coefficient, varying within the range of 10^−32^ cm^6^/s to 10^−14^ cm^6^/s. All parameters exhibited a pronounced variation up to an $B_{Auger}$ coefficient of 10^−24^ cm^6^/s. With an increase in the Auger recombination coefficient from 10^−24^ cm^6^/s to 10^−14^ cm^6^/s, as illustrated in Figure 10a–d, all PV parameters of the solar cell experienced a notable reduction. A substantial degradation in the J−V characteristics is observed when the $B_{Auger}\;$ coefficient exceeds $B_{Auger,n}$ 10^−24^ cm^6^/s: $V_{OC}$ decreased from 0.681 V to 0.377 V, $J_{SC}$ declined from 29.22 mA/cm^2^ to 8.54 mA/cm^2^, FF decreased from 48.46% to 37.79%, and η diminished from 19.36% to 2.43%, respectively (in Figure 10a–d). When $B_{Auger}\;$ becomes dominant, the recombination rate increases, and carriers are destroyed very quickly within the absorber before they can be collected. Carrier lifetime and diffusion length are severely reduced, physically limiting the carriers’ ability to reach the joint area. This rapid decrease in carrier density reduces quasi-Fermi level separation, leading to a sharp decrease in *V_oc_*, while the shortened diffusion length reduces the probability of photocarrier collection, resulting in a significant decrease in *J_sc_*.

As depicted in Figure 10c, for an Auger recombination coefficient of $B_{Auger}{=10}^{-30\;},\;10^{-29},\;10^{-28}$ cm^6^/s, the charge recombination process remains comparatively minimal relative to the charge generation rate. For $B_{Auger}{=10}^{-30\;},\;10^{-29},\;10^{-28},\;10^{-27},\;10^{-26}$ cm^6^/s, Auger electron recombination occurring at the edges of the depletion region was characterized by values of 1.36 × 10^20^, 1.16 × 10^21^, 1.22 × 10^22^, 1.24 × 10^23^, 7.24 × 10^23^ (1/cm^3^·s), respectively. After $10^{-27}$ cm^6^/s, the Auger electron capture coefficient exceeded the amount of charge generation.

#### 3.2.3. Effect of the Defect Density in the Absorber Layer

In CTS thin film, Cu deficiency, Sn irregularities, and antisite defects (such as Cu_Sn_ and Sn_Cu_) create dense trap levels within the band gap. Irregularities in the crystal structure include secondary phases such as Cu_2_Sn and SnS_2_, and phase and grain boundaries act as both potential barriers and scattering centers for carriers. Furthermore, the strain within the absorber sample, along with the consistency of pinholes and cracks forming during thin film production and an increased density of grain boundaries, leads to the development of defects and trap sites, ultimately shortening the carrier lifetime. The degradation rate amplifies with a reduction in the stability of the sample. The heightened degradation results in an increased defect density, which may subsequently facilitate the predominance of SRH recombination in the absorber layer [43,44]. The impact of defect density can be described using the SRH model, where electrons transition between conduction and valence bands via intermediate energy states (localized states) introduced within the band gap by defects [45].

(8) $$R=\frac{np-n_{i}^{2}}{\tau_{p}\left(n+N_{C}e^{\frac{\left(E_{g}-E_{t}\right)}{k_{B}T}}\right)+\tau_{n}\left(p+N_{V}e^{\frac{\left(E_{t}\right)}{k_{B}T}}\right)}$$

The electron and hole mobility are denoted by $\mu_{n,p}$, while q represents the carrier charge. The equations illustrate that a rise in defect density in the semiconductor leads to improved carrier recombination, which in turn shortens the carrier lifetime and negatively impacts the performance of the solar cell. The variables p and n correspond to the concentrations of free holes and electrons, respectively, while $E_{t}$ indicates the energy level at which defect-related trap states are positioned. The defect density, as defined in Equation (8), serves as a critical parameter for characterizing the carrier recombination rate (R). The electron and hole lifetimes, denoted by $\tau_{n}$ and $\tau_{p}$, respectively, are quantifiable via the expression outlined in Equation (9) [46].

(9) $$\tau_{n,p}=\frac{1}{\sigma_{n,p}.\nu_{th}.N_{t}}$$

$\sigma_{n}$ and $\sigma_{p}$ characterize the effective cross-sectional areas for electron and hole scattering events, respectively. The trap defect density is represented by $N_{t}$, and $\nu_{th}$ signifies the thermal velocity. The correlation between carrier lifetime and diffusion length is formalized in Equation (10) [46].

(10) $$L_{n,p}=\sqrt{\frac{{µ}_{n,p}k_{B}T}{q}\tau_{n,p}}$$

**Figure 11:**
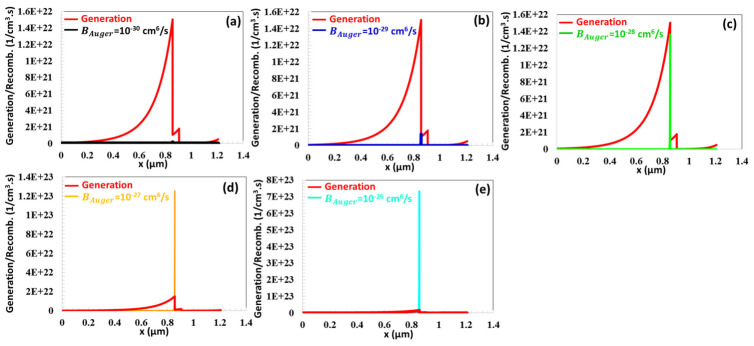
(**a**–**e**) Generation and recombination profiles versus position (x) of the CTS TFC for $B_{Auger}$ varying from 10^−30^ to 10^−26^ cm^6^ s^−1^.

**Figure 12:**
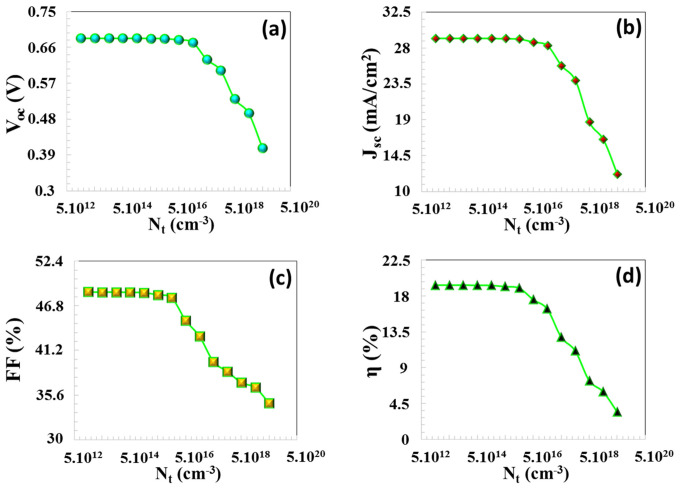
(**a**) $V_{OC}$, (**b**) $J_{SC}$, (**c**) FF, (**d**) η PV parameters depending on $N_{t}$ (cm^−3^) in the CTS absorber layer.

The electron and hole mobilities are denoted by $\mu_{n}$ and $\mu_{p}$, respectively, with q representing the carrier charge. The derived equations demonstrate that an increase in semiconductor defect density results in an enhanced recombination of charge carriers, which reduces their lifetime and leads to a deterioration in the performance of the solar cell. Figure 11a–d reveals that PV parameters remained relatively unaffected within the trap defect density range from $N_{t}$ = 1.10^13^ to 5.10^15^ cm^−3^. At low Nt values, these trap levels are low, and the carriers have a high mean free path and lifetime. Therefore, the generated electron-hole pairs are largely decomposable and can be transported to the contacts with the contribution of the electric field. In this region, the solar cell operates efficiently with a balance of diffusion and drift. Nevertheless, after a defect density of 3.10^13^ cm^−3^ value, while FF exhibits some increase, a decline in other PV parameters was noted. Specifically, as the trap defect density was increased from 1.10^16^ cm^−3^ to 5.10^19^ cm^−3^, $V_{OC}$,${\;J}_{SC}$, FF, and efficiency decreased from 0.682 V to 0.407 V, from 29.17 mA/cm^2^ to 12.13 mA/cm^2^, from 48.16% to 34.57%, and from 19.17% to 3.41%, respectively, as seen in Figure 12. As a consequence, there is a corresponding decline in $V_{OC}$, ${\;J}_{SC}$, FF, and efficiency values. As $N_{t}$ increases, defect levels form intermediate locations for electrons and holes; an electron is first captured at this level, then recombines with a hole. The distance between semi-Fermi levels decreases and is directly observed as a drop in Voc. The lifetime of charge carriers subjected to recombination is reduced, defects also act as scattering centers, mobility is indirectly reduced, and the drift speed of the carriers decreases, thus causing a decrease in ${\;J}_{SC}$ and FF values.

**Figure 13:**
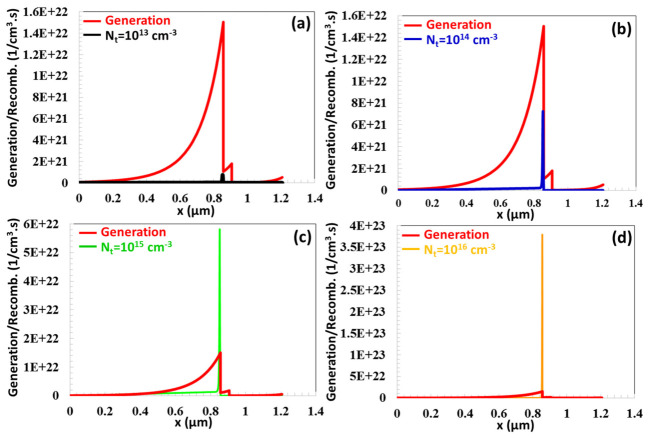
(**a**–**d**) Generation–recombination profile as a function of position for the CTS TFC at absorber layer defect densities ranging from 10^13^ cm^−3^ to 10^16^ cm^−3^.

Figure 13 shows the generation–recombination characteristics as a function of position (x) within the active layer of the solar cell, showing that for defect densities $N_{t}$ in the absorber layer ranging from 1.10^13^ cm^3^ and 5.10^19^ cm^3^, SRH recombination rate remains lower than the generation rate up until $N_{t}$ = 10^15^ cm^−3^. After $N_{t}$ = 10^15^ cm^−3^, SRH recombination rate escalates considerably, adversely impacting PV performance. An increase in $N_{t}$ results in a reduction of the minority carrier diffusion length (*L_n_*), and a concomitant decrease in their lifetime ($\tau_{n}$), as indicated in Table 2. Notably, as $N_{t}$ risen from 10^13^ cm^−3^ to 10^16^ cm^−3^, both $L_{n}$ and $\tau_{n}$ diminished significantly, with $L_{n}$ dropping from 7.7 × 10^1^ µm to 2.4 × 10^0^ µm and $\tau_{n}$ reducing from 10^4^ ns to 10^1^ ns. This decrease in carrier parameters resulted in diminished charge accumulation within the solar cell, which in turn negatively impacted all PV performance metrics.

### 3.3. Effect of the Operating Temperature

**Figure 14:**
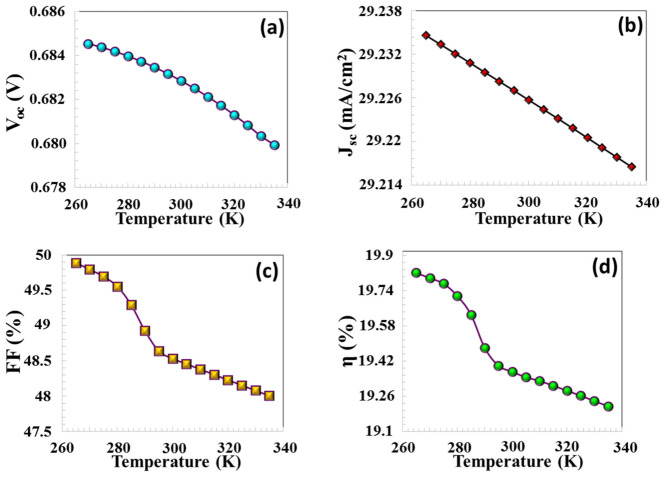
(**a)** $V_{OC}$, (**b**) $J_{SC}$, (**c**) FF, (**d**) η PV parameters depending on the operating temperature.

The performance of solar cells is significantly affected by the operating temperature. As demonstrated in Figure 14a–d, when the temperature was elevated from 200 K to 400 K, a reduction in $V_{OC}$, $J_{SC}$, FF, and efficiency was observed. An upsurge in temperature leads to a higher intrinsic carrier concentration and a corresponding reduction in the semiconductor energy gap. Consequently, the rise in reverse saturation current density leads to a decline in ($V_{OC}$). An increase in temperature results in band gap narrowing (negative ${dE}_{g}/dt$), which facilitates electron–hole recombination between the conduction and valence bands of the semiconductor and consequently increases the dark current of the TFC. The correlation between the operating temperature, the semiconductor’s energy band gap, and $V_{OC}$ is mathematically represented by the following equation [47].

(11) $$\frac{d(V_{oc})}{dT}=\frac{\left(V_{oc}-\frac{E_{g}}{q}\right)}{T}$$

Elevated temperatures supply thermal energy to the electrons, resulting in their excitation to higher energy states. Consequently, the electrons are rapidly excited into the conduction band, which indirectly leads to a decline in the energy gap. As outlined in Equation (11), an increase in temperature results in a reduction of both $V_{OC}$ and the band gap.

### 3.4. The Effect of the Series and the Shunt Resistance

The predominant parasitic resistances in TFCs are the series resistance ($R_{S}$) and the shunt resistance ($R_{sh}$). $R_{S}$ denotes the resistance encountered between the semiconductor and the metal contact, arising from the flow of current across the interface of the two layers, as well as other internal resistances within the device [48]. Moreover, $R_{S}$ is influenced by factors such as surface grain boundaries, strain, interface trap states, and the resistance associated with the thin film surface. $R_{sh}$ resistance arises from defects introduced during thin film production, such as crack or pinhole formation, losses due to unintended alternating current pathways, and leakage currents at the junction area [49].

The influence of $R_{S}$ and $R_{sh}$ resistances on the PV parameters is given by Equation (12) [33]:

(12) $$I=I_{ph}-I_{o}\left[exp\left(\frac{q\left(V+IR_{s}\right)}{nkT}\right)-1\right]-\frac{V+IR_{s}}{R_{sh}}$$

I denotes the output current, *I_o_* represents the saturation current, $I_{ph}$ is the photocurrent, V  indicates the voltage, q refers to the elementary charge, k  is the Boltzmann constant, n is the ideality factor, and T stands for the temperature.

**Figure 15:**
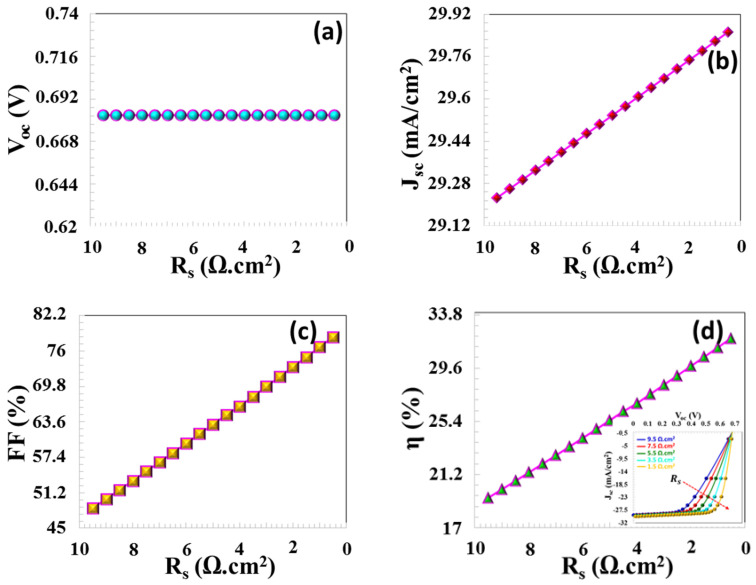
(**a**) $V_{OC}$, (**b**) $J_{SC}$, (**c**) FF, and (**d**) η  PV parameters.

In this theoretical analysis, the value of $R_{S}$ was varied from 9.5 Ω·cm^2^ to 0.5 Ω·cm^2^, while $R_{sh}$ was adjusted from 5 × 10^−1^ Ω·cm^2^ to 3 × 10^3^ Ω·cm^2^. The reduction in series resistance ($R_{S}$) did not lead to a substantial variation in $V_{OC}$; however, a notable enhancement in $J_{SC}$, FF, and overall efficiency was observed, as demonstrated in Figure 15a–d. Elevated series resistance could have contributed to a decrease in $J_{SC}$, as it likely hindered the effective transport of charge carriers. When $R_{S}$ was decreased from 9.5 Ω·cm^2^ to 0.5 Ω·cm^2^, the values of $J_{SC}$, FF, and η increased significantly, rising from 29.22 mA/cm^2^ to 29.85 mA/cm^2^, from 48.53% to 78.44%, and from 19.37% to 31.96%, respectively. These values are consistent with PV data from modeled CTS solar cells reported in the literature [33,50,51,52,53,54]. Specifically, the fact that metal contacts exhibit more ohmic behavior due to reduced resistance, and that the reduction in surface resistance in thin films facilitates charge transfer between the two layers, leads to an increase in PV values. An increase in shunt resistance results in a reduction of the current flowing through the shunt path, which subsequently leads to an enhancement in both $V_{OC}$ and $J_{SC}$. The increase in shunt resistance leads to an improvement in both V_ox_, $J_{SC}$, FF and η as illustrated in Figure 15a–d. In the case of an infinite shunt resistance, the output voltage can be represented by the following equation:

(13) $$V=\frac{kT}{q}ln\left(\frac{I_{sc}}{I_{o}}+1\right)$$

**Figure 16:**
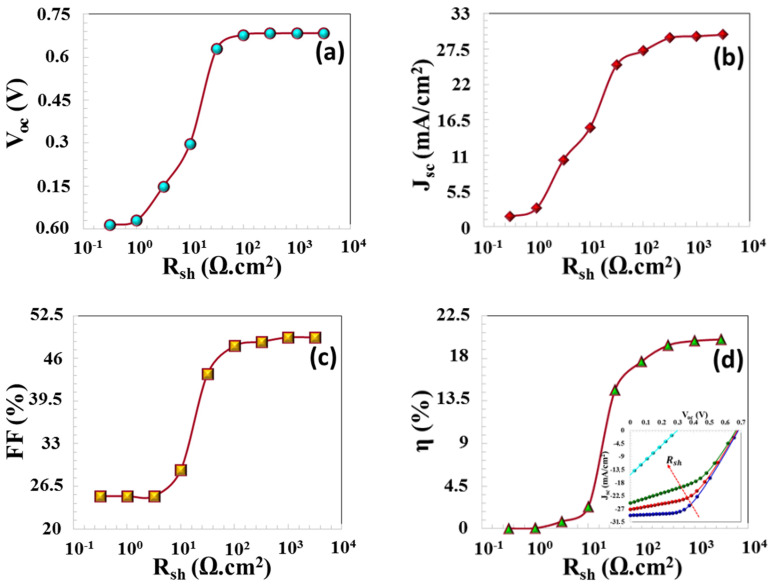
(**a**) $V_{OC}$, (**b**) $J_{SC}$, (**c**) FF, and (**d**) η  PV parameters.

The reverse saturation current ($I_{o}$) has an adverse impact on the output voltage. This relationship is mathematically represented in Equation (13). The presence of a leakage current within the junction region, which can be attributed to defects introduced during the thin film fabrication process. Consequently, a reduction in the shunt resistance led to a corresponding decrease in all PV parameters of solar cell, as illustrated in Figure 16a–d.

According to Table 3, the calculated and optimized PV results of solar cells modeled with SCAPS, as reported in the literature and in this study, are somewhat higher. Because several important physical and structural effects observed in experimentally fabricated solar cells cannot be fully modeled due to their one-dimensional approach in SCAPS-1D software. Morphological features such as lateral current flow, grain boundaries, local defects, cracks, pinholes, and surface roughness are neglected, and all layers are assumed to be homogeneous despite the presence of composition gradients and doping fluctuations in real devices. Complex defect distributions and multi-level trap systems are simplified, while optical effects such as light scattering and wave optics, as well as mechanical and thermal effects, are not included. At the interfaces, chemical reactions, diffusion, and interphase formations are not treated in detail, and interface recombination is typically represented by simplified trap densities. Similarly, non-ideal metal/semiconductor contact effects such as Fermi level pinning, contact resistance, and interface irregularities are not fully considered. As a result, the solar cell’s operating performance, whether weak or improved, has been demonstrated, and these values are consistent with the literature [55,56,57]. The SCAPS-1D program reliably analyzes carrier transport, recombination mechanisms, and energy band alignment using a physics-based drift-diffusion model, enabling a clear understanding of the fundamental processes that determine device performance. One of the program’s advantages is its ability to provide systematic parametric analysis and optimization of variables such as layer thickness, band gap, electron affinity, doping density, and defect parameters. Furthermore, its ability to comparatively examine different materials and layer structures allows for in-depth evaluation from a band alignment and interface engineering perspective; this plays a critical role in understanding the impact of structural enhancements, such as BSF, on device performance.

### 3.5. The Effect of Different BSF Layers

The heavily doped *p*-type semiconductors, called BSF layers, are placed between the back contact and the absorber layer in solar cells and play a significant role in the efficiency of solar cells [64]. When an electron in CTS semiconductor reaches the rear surface of TFC, it is often captured, rendering it unable to contribute to the current flow. BSF layer acts like a quasi-ohmic contact and forms barrier height or built in voltage as seen in the band diagram in Figure 16 [38,65]. The BSF layer, by applying a high electric field to electrons coming from the conduction band of CTS, pushes the electrons back to the depletion region. Thus, the minority charge carriers (electrons) contribute to charge accumulation at the boundary of the depletion region without undergoing recombination in the back contact region and enhance $J_{SC}$ value. In particular, ultra-TFCs, such as CTS solar cells with absorber layer thicknesses less than 1000 nm, may have somewhat low $V_{OC}$ values. In such thin film solar cells, BSF layers improve $V_{OC}$ value by preventing the recombination of minority charge carriers at the back contact [66]. If there is a coherent band alignment between BSF and CTS semiconductor layers, holes in the valence band of CTS readily migrate to the BSF layer without undergoing recombination, thus contributing to $V_{OC}$ and $J_{SC}\;$ [38].

**Figure 17:**
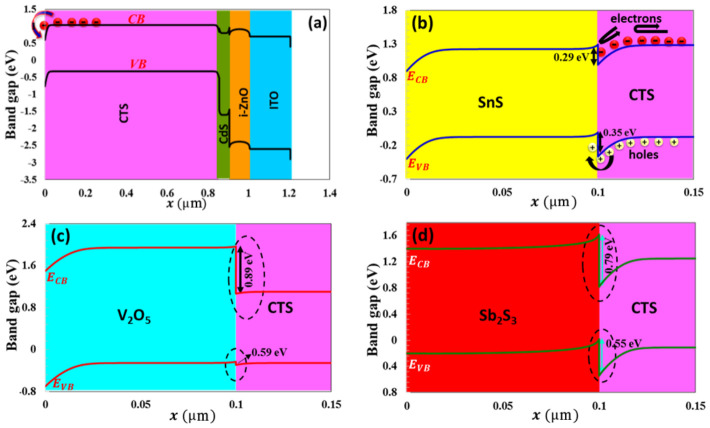
The band diagram of Au/CTS/CdS/i-ZnO/ITO with (**a**) no BSF and (**b**) SnS, (**c**) V_2_O_5_ and (**d**) Sb_2_S_3_ BSF layer.

**Figure 18:**
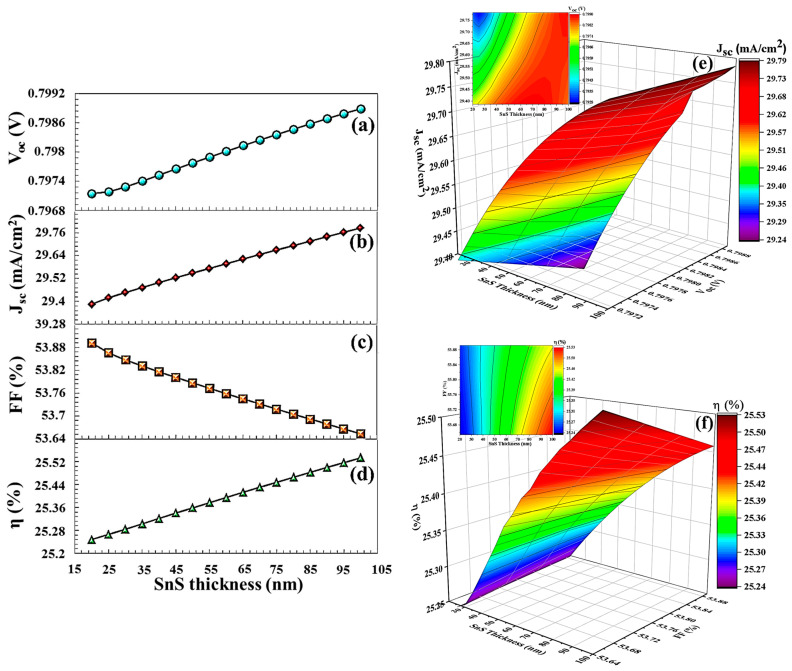
(**a**–**d**) $V_{OC}$, $J_{SC}$, FF,  η  PV parameters, (**e**,**f**) the 3D contour plots, depending on SnS BSF layers thickness.

The VB maximum of the BSF layer must exhibit precise alignment with the VB maximum of the absorber semiconductor layer to ensure optimal electronic interaction and efficiency. This alignment minimizes the energy barrier for hole transport from the CTS absorber layer to the back contact via BSF, thereby enhancing charge carrier mobility and improving overall device performance [67]. Efficient hole transport is achieved through optimal energy level alignment, which minimizes both energy loss and the likelihood of recombination. As shown in the band diagram in Figure 17a, in a BSF-free solar cell, there is no barrier height between the back contact and the active layer, allowing minority charge carriers to easily pass from the active layer to the metal contact. Without BSF, there is no energy selectivity between the back contact and the CTS absorber. Electrons and holes reach the back surface by diffusion. High-speed surface recombination occurs on the back surface. Since the carrier flow is not controlled by drift, the system operates in the diffusion-limited recombination region. This leads to recombination, which somewhat degrades the solar cell’s performance. In this study, in order to enhance the efficiency of the solar cell, SnS, PbS, V_2_O_5_, Sb_2_S_3_, and BSF layers were used, and their physical parameters are given in Table 2. As seen in Figure 17b, the SnS BSF layer formed a barrier height, limiting the movement of minority charge carriers to the back region and redirecting them back to the deposition region. At the SnS interface, a 0.29 eV electron barrier occurs in the conduction band and a 0.35 eV hole alignment occurs in the valence band. Valence band alignment forms a low-resistance path for holes and increases electron–hole separation, thus forming strong carrier selectivity. The parameters $V_{OC}$, $J_{SC},\;FF$, and η  of the solar cell for 20 nm thickness of SnS increased significantly to 0.7971 V, 29.38 mA/cm^2^, 53.89%, and 25.24%, respectively (in Figure 18).

**Figure 19:**
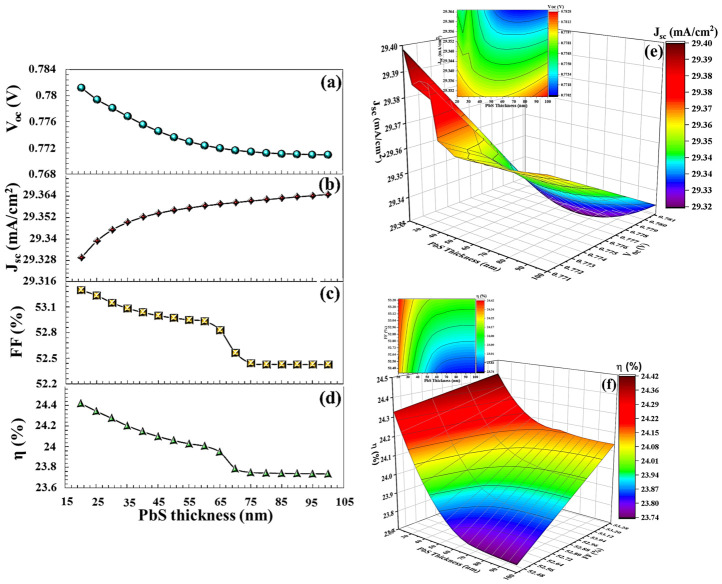
(**a**–**d**) $\;V_{OC}$, $J_{SC}$, FF,  η  PV parameters, (**e**,**f**) the 3D contour plots depending on PbS BSF layers thickness.

**Figure 20:**
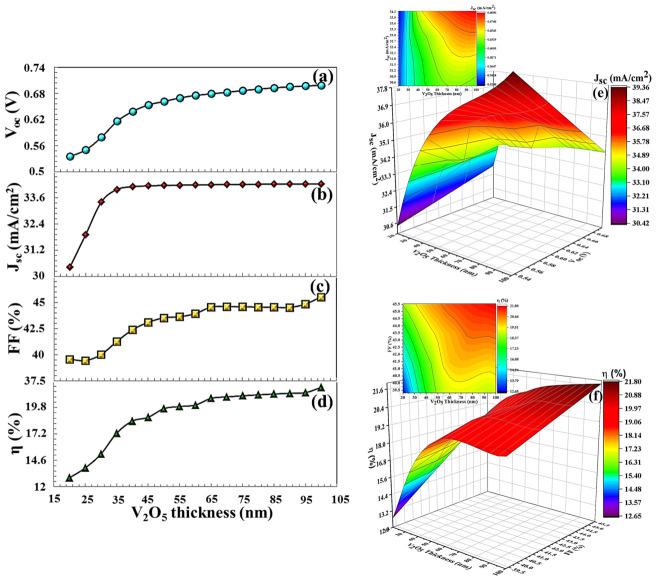
(**a**–**d**) $V_{OC}$, $J_{SC}$, FF,  η  PV parameters, (**e**,**f**) the 3D contour plots depending on the V_2_O_5_ BSF layers thickness.

**Figure 21:**
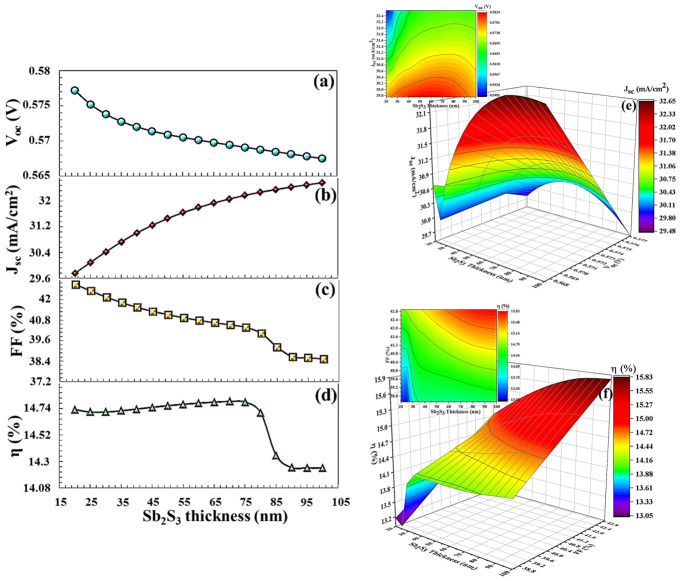
(**a**–**d**) $V_{OC}$, $J_{SC}$, FF,  η  PV parameters, (**e**,**f**) the 3D contour plots depending on Sb_2_S_3_ BSF layers thickness.

For PbS BSF (20 nm thickness), which has the same band gap and electron affinity as SnS, $V_{OC}$ = 0.781 V, $J_{SC}$ = 29.329 mA/cm^2^, FF = 53.29%, and η = 24.42% (in Figure 19). For V_2_O_5_ and Sb_2_S_3_ (20 nm thickness), the efficiency parameters showed a decrease to 12.89% and 14.79%, respectively, in Figure 20 and Figure 21. As seen in the band diagram in Figure 16, although BSF layers with this high band gap exhibit high barrier height, their low electron affinities cause electrons to easily recombine on this surface, leading to a decrease in performance. Specifically, mismatched band alignment in the valence band of V_2_O_5_ can cause recombination during hole transition [67]. The lower acceptor carrier density of Sb_2_S_3_ has reduced the likelihood of heavy doping, potentially decreasing the electrical field that would repel minority charge carriers within the absorber layer, thereby reducing charge accumulation and current. Surfaces with low electron affinity often have a high work function. This makes it difficult for carriers (especially electrons) to move across the surface. The high work function prevents carriers from interacting smoothly with the back electrodes. Furthermore, in the case of V_2_O_5_, CB (0.89 eV), and VB (0.59 eV), alignments are more incompatible with the CTS thin film. For large ΔE_c_, electrons are completely blocked, while for large ΔE_v_, holes also hit the energy wall. This bidirectional barrier makes the interface a charge accumulation region. Carriers can accumulate at the interface, increasing local charge density, distorting the electric field in the opposite direction, and enhancing recombination. For Sb_2_S_3_ BSF (CB (0.79 eV) and VB (0.55 eV)), an electron barrier exists but is not as optimized as in SnS. Valence band alignment is not entirely ideal; electrons are partially repelled, but carrier dissociation is incomplete because the local electric field remains weak, and the diffusion component becomes dominant again.

As the thickness of BSF increases, electrons and holes have a longer distance between them, and the probability of recombination decreases in this process [68]. A thick BSF layer evenly orients the carriers, allowing more carriers to associate with the electrode. Thick BSF layers passivation more effectively on surface defects. Passivation prevents carrier losses by neutralizing active transition levels on the surface. Increasing the thickness of the BSF layer creates a stronger electrical field on the back surface. This electrical field moves the carriers in the correct direction. As the thickness of BSF layer increases, more light can be reflected from the back surface. This allows more light to return to the active area, thus promoting more efficient light absorption by the cell. Therefore, increasing the thickness of SnS and V_2_O_5_ layers increased the efficiency of solar cell to 25.53% and 21.79%, respectively. In particular, after a V_2_O_5_ thickness of 50 nm, an efficiency (19.69%) was obtained higher than that in a solar cell without BSF (19.37%) in Figure 20.

**Figure 22:**
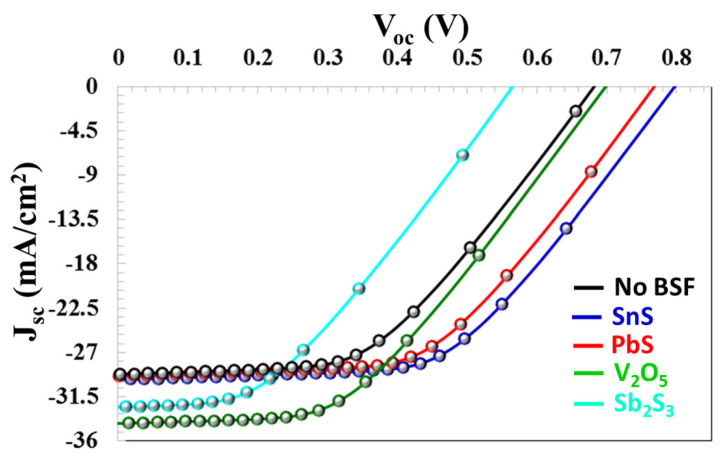
J−V characteristic depending on no BSF and SnS, PbS, V_2_O_5_, and Sb_2_S_3_ BSF layer thickness.

As a result, the band gap, electron affinity, acceptor carrier density, and thickness of BSF layer are of great importance in determining the efficiency of the solar cell. Compared to other layers, the SnS BSF layer exhibited ideal BSF characteristics, as seen in the J−V characteristic in Figure 22. The low cost, environmental friendliness, and ease of production of the SnS layer make it highly advantageous for use as a BSF layer in CTS solar cells. So, SnS BSF’s high electroaffinity forms a favorable conduction band offset relative to the absorber side by pulling the conduction band down, preventing minority carrier electrons from reaching the back metal contact and redirecting them back into the absorber. This reduces back-surface recombination, increases carrier lifetime, and improves both *V_oc_* and *J_sc_*. At the same time, the appropriate χ value does not impair hole transport by preventing excessive barrier formation; thus, the system achieves balanced selectivity. A high dielectric permittivity of SnS BSF allows the material to carry the electric field better and results in a more uniform distribution of the charge region. Coulombic scattering and potential fluctuations at the interface weaken, and local electric fields are created by defect and trap levels. Thus, carriers undergo less scattering, and interface recombination is suppressed. As a result, carrier transport becomes more stable, and FF increases. High acceptor defect density (high *p*-type doping) forms a strong built-in electric field in the BSF layer. This strong field increases band bending at the absorber/BSF interface and forms a more effective repulsive force, especially for electrons. That is, electrons have to climb upwards in energy as they approach the back surface and therefore return. At the same time, high doping provides a low-resistance conduction path for holes (quasi-ohmic contact) so that the majority carriers are easily transported to the back contact. Thus, both recombination is reduced and the series resistance is decreased, thus increasing FF and efficiency. Due to all these factors, the SnS BSF-containing CTS solar cell exhibited the highest PV performance.

## 4. Conclusions

In this study, PV performance of an Au/BSF/CTS/CdS/i-ZnO/ITO TFSC was systematically investigated using SCAPS-1D simulation software. The effect of interface defect density, recombination mechanisms, absorber defect density, operating temperature, parasitic resistances, and different BSF layers on the device performance was comprehensively analyzed. The simulation results revealed that the interface defect density plays a crucial role in determining PV characteristics of the CTS solar cell. When the interface defect density increased from 1 × 10^12^ cm^−3^ to 1 × 10^16^ cm^−3^, the $V_{OC\;}$ decreased from 0.682 V to 0.456 V, while the $J_{SC\;}$ declined from 29.22 mA/cm^2^ to 27.12 mA/cm^2^. Consequently, the PCE dropped significantly from 19.14% to 7.47%, indicating the strong influence of recombination centers at the interface. The effects of radiative and $B_{Auger}\;$ were also evaluated. It was observed that the solar cell maintained stable PV performance for $B_{r}\;$ between 10^−15^ cm^3^/s and 5 × 10^−10^ cm^3^/s. However, further increases in the recombination coefficient resulted in severe degradation of the device performance, reducing the efficiency from 19.12% to 3.66%. Similarly, when the $B_{Auger}\;$ coefficient exceeded 10^−24^ cm^6^/s, a remarkable deterioration in PV parameters was observed.

The defect density within the CTS absorber material was found to be another critical factor affecting the performance of the device. As the defect density increased from 10^16^ cm^−3^ to 5 × 10^19^ cm^−3^, $V_{OC}$, $J_{SC}$, FF, and efficiency decreased from 0.682 V to 0.407 V, 29.17 mA/cm^2^ to 12.13 mA/cm^2^, 48.16% to 34.57%, and 19.17% to 3.41%, respectively. These results indicate that higher defect densities significantly enhance carrier recombination and reduce the minority carrier lifetime. The operating temperature also exhibited a noticeable effect on PV characteristics. When the temperature increased from 200 K to 400 K, a gradual reduction in $V_{OC}$, FF, and overall efficiency was observed because of energy gap reduction and increased recombination processes. Furthermore, the impact of parasitic resistance was investigated. Reducing the series resistance from 9.5 Ω·cm^2^ to 0.5 Ω·cm^2^ resulted in a remarkable improvement in the device efficiency from 19.37% to 31.96%, while increasing the shunt resistance significantly enhanced the overall device stability and PV performance. Finally, the role of different BSF layers was examined in order to suppress the recombination of minority carriers at the back contact. Among the investigated BSF materials, BSF layer of SnS demonstrated the best performance due to its favorable band alignment with the CTS absorber layer. With an optimized SnS thickness of 20 nm, PV parameters reached $V_{OC}$ = 0.797 V, $J_{SC}$ = 29.38 mA/cm^2^, FF = 53.89%, and a maximum PCE of 25.24%.

## Figures and Tables

**Table 2:** Variation of the electron/hole diffusion length and electron/hole lifetime as a function of defect density in the CTS absorber layer.

*N_t_* (cm^−3^)	Electron Diffusion Length (*L_n_*) (μm)	Hole Diffusion Length (*L_p_*) (μm)	Electron Lifetime (*τ_n_*) (ns)	Hole Lifetime (*τ_p_*) (ns)
10^13^	7.7 × 10^1^	2.5 × 10^1^	10^4^	10^4^
10^14^	2.4 × 10^1^	8.0 × 10^0^	10^3^	10^3^
10^15^	7.7 × 10^0^	2.5 × 10^0^	10^2^	10^2^
10^16^	2.4 × 10^0^	8.0 × 10^−1^	10^1^	10^1^

**Table 3:** Comparison of PV parameters of CTS thin-film solar cells modeled with the SCAPS-1D program reported in the literature.

Solar Cell	*V_oc_* (V)	*J_sc_* (mA/cm^2^)	FF (%)	*η* (%)	REF
FTO/CTS/ZnS/Ag	0.425	24.82	78.18	8.25	[58]
p-CTS/n-CdS/i-ZnO/n-ZnO:Al	0.58	48.71	78.13	22.35	[59]
Mo/CTS/ZnS/ITO	0.795	34.19	62.68	17.05	[60]
Mo/CTS/CdS/ITO	0.794	34.01	61.17	16.53	[60]
iZnO/CdS/p-CTS	0.712	35.00	-	20.3	[61]
Au/CTGS/CdS/i-ZnO/AZO	0.788	41.09	85.30	27.30	[33]
n-ZnSe/p-CTS	0.602	32.3	80.58	30.00	[50]
Mo/CGS/CTS/ZnSe/Ti	0.600	32.39	80.58	17.69	[54]
Al:ZnO/ZnO/CdS/CTS/Mo	0.827	35.23	77.32	22.56	[62]
FTO/CdS/CTS	0.753	46.5	86.88	31.8	[63]
FTO/CdSe/CTS	0.781	47.1	86.90	32.0	[63]
SLG/Mo/CTS/ZnS/ITO/Al	1.091	24.6	75.23	20.3	[36]
Au/CTS/CdS/i-ZnO/ITO	0.682	29.85	78.44	31.96	In this study

## Data Availability

The data presented in this study is available on request from the corresponding author.
